# EXAMINATION OF TRUST IN THE HEALTH CARE SYSTEM AMONG OLDER IMMIGRANTS RESIDING IN THE MIDWEST

**DOI:** 10.1093/geroni/igz038.1903

**Published:** 2019-11-08

**Authors:** Robyn Husa, Hisako Matsuo, Jennifer Hale Gallardo, Lisa Willoughby

**Affiliations:** 1 Saint Louis University, St. Louis, Missouri, United States; 2 CINDRR-Gainsville, Gainesville, Florida, United States

## Abstract

Ethnic minority populations, such as immigrants, have demonstrated lower levels of trust in the health care system and their health care providers compared with non-migrant populations (Navaza et al., 2012; Renzaho, Polonsky, McQuilten, & Waters, 2013). This medical mistrust may adversely influence older adult immigrants’ use of and satisfaction with health services (Hong et al., 2018a; Jang, Kim, & Chiriboga, 2005). Thus, the current project aimed to characterize influences of medical mistrust (healthcare system and healthcare providers) in older adult immigrant populations living in the United States of America (U.S.). We interviewed 142 older adult immigrants and refugees (aged 60+ years) who identified as Bosnian, Chinese, Indian, Korean, Latino, and Vietnamese about their perceptions on living in the U.S., of the healthcare system, and healthcare utilization as a part of the Successful Aging among Immigrants in Midlife (SAIM) project. Linear regression models predicting trust in the healthcare system and trust in healthcare providers were tested with healthcare knowledge (measured with a single item about flu shots) , acculturation, length of time in the U.S. , and age as predictors. We found that older age and healthcare knowledge was predictive of higher levels of trust in healthcare providers for Chinese. Although healthcare knowledge was predictive of trust in the health care system for the Chinese participants, greater length of time and higher acculturation were associated with higher trust in the healthcare system among Indian participants. The implications of the different predictive variables in each of the hypothesized models will be discussed.

